# The early identification of risk factors on the pathway to school dropout in the SIODO study: a sequential mixed-methods study

**DOI:** 10.1186/1471-2458-12-1033

**Published:** 2012-11-27

**Authors:** Marie-José Theunissen, Ilse Griensven van, Petra Verdonk, Frans Feron, Hans Bosma

**Affiliations:** 1 CAPHRI, Department of Social Medicine, Maastricht University, Maastricht, The Netherlands; 2 GGD Brabant Zuidoost, Eindhoven, The Netherlands; 3 EMGO Institute for Health and Care Research, Department of Medical Humanities, VU University Medical Center, Amsterdam, The Netherlands

**Keywords:** School dropout, Risk profiles, Pathway, Symptoms, Life course, ICF model, Socioeconomic status, Gender, Public health, Social exclusion

## Abstract

**Background:**

School dropout is a persisting problem with major socioeconomic consequences. Although poor health probably contributes to pathways leading to school dropout and health is likely negatively affected by dropout, these issues are relatively absent on the public health agenda. This emphasises the importance of integrative research aimed at identifying children at risk for school dropout at an early stage, discovering how socioeconomic status and gender affect health-related pathways that lead to dropout and developing a prevention tool that can be used in public health services for youth.

**Methods/design:**

The SIODO study is a sequential mixed-methods study. A case–control study will be conducted among 18 to 24 year olds in the south of the Netherlands (n = 580). Data are currently being collected from compulsory education departments at municipalities (dropout data), regional public health services (developmental data from birth onwards) and an additional questionnaire has been sent to participants (e.g. personality data). Advanced analyses, including cluster and factor analyses, will be used to identify children at risk at an early stage. Using the quantitative data, we have planned individual interviews with participants and focus groups with important stakeholders such as parents, teachers and public health professionals. A thematic content analysis will be used to analyse the qualitative data.

**Discussion:**

The SIODO study will use a life-course perspective, the ICF-CY model to group the determinants and a mixed-methods design. In this respect, the SIODO study is innovative because it both broadens and deepens the study of health-related determinants of school dropout. It examines how these determinants contribute to socioeconomic and gender differences in health and contributes to the development of a tool that can be used in public health practice to tackle the problem of school dropout at its roots.

## Background

Good education increases life expectancy, reduces the burden of illness, delays the consequences of aging, decreases unhealthy behaviours and reduces health inequalities [1]. Some have even argued that investments in education may save more lives than advances in medical technologies [2]. However, almost 14% of all 18 to 24 year olds in Europe finish their education without a certificate or attain only a low level of education that is of little use on the labour market [3]. School dropout is likely to have important implications for the mental and physical health status of a dropout. Health and developmental problems might also be implicated in the pathways leading to dropout. Despite its likely embedding in the discussion on socioeconomic inequalities in health, the issue of school dropout has mostly been neglected on the agenda of public health researchers and practitioners. To develop effective interventions, integrative research is needed into school dropout, and particularly its health-related determinants along the life course.

Current evidence on determinants can be grouped into family-related determinants (e.g. living in a single-parent family) [1,4,5], school-related determinants (e.g. little concern in school policies) [1,4-8] and pupil-related determinants (e.g. life-styles and personality) [1,5,9,10]. This evidence is, however, fragmented and has not yet been considered in a more integrative approach that acknowledges the life-course and developmental pathways that lead to school dropout [7,11]. This study uses a life-course, integrative focus to assess these pathways and proposes a mixed-methods design in order to broaden and deepen the insight into these pathways. Including early life will also make it possible to identify determinants that can be used in interventions aimed at preventing young people from dropping out of school.

We particularly expect socioeconomic status, sex (male, female) and gender (masculinity, femininity) to be important fundamental determinants in this study [1,5,12,13]. Girls tend to graduate faster and with higher grades than boys [4,14]. Boys more often engage in deviant behaviours and are more likely to drop out of school than girls [13,15]. Deviant behaviours and dropping out of school might give boys respect from their peers, prove their masculinity, and make them feel ‘too cool for school’ [16-20]. The risk of dropout among girls, on the other hand, may be more strongly related to the tendency to internalise problems involving temperamental inhibition and depressive symptoms [21-24]. Poor socioeconomic conditions during a child’s upbringing are another fundamental background characteristic that is related to school dropout [12,13] as well as poor health in children and adolescents [25]. Poor health may even prove to be an under-studied mediator in the intergenerational reproduction of socioeconomic differences [9]. Furthermore, the fact that early school leavers often have a poor health status [26] emphasises the importance of addressing early health problems and school dropout in the generational and intergenerational reproduction of socioeconomic inequalities in health. Our study aims to provide detailed insight into how gender and socioeconomic status might set children on a pathway leading to school dropout.

The aim of the SIODO (stay in or drop out) study is to provide insight into the life-course pathways leading towards school dropout and the socioeconomic and gender differences found in these pathways. Exploring early health conditions in socioeconomic, environmental, psychosocial and personality- and gender-related contexts will also provide input for developing a tool that can be used at an early stage to identify children that are at risk of dropping out of school. This article presents the design of the SIODO study.

## Methods/design

**Figure 1:**
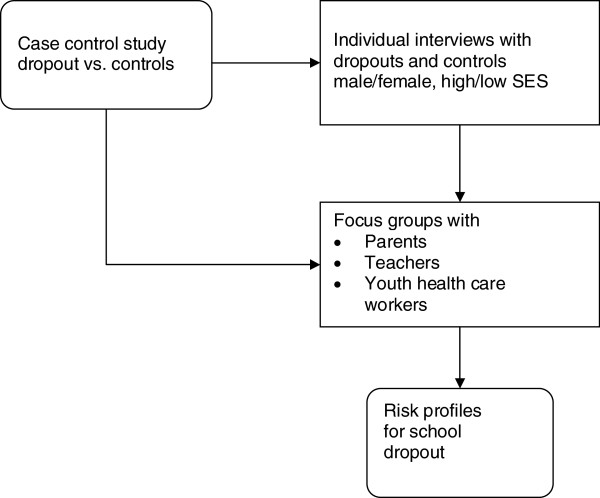
**SIODO flowchart.** The results of the case–control study will provide input for the individual interviews and for the focus groups, which in turn will help to understand the data and gain further insight into pathways leading to school dropout. The results from the individual interviews will be discussed in the focus groups.

The SIODO study is a sequential mixed-methods study (Figure 1) [27]. We designed an unmatched case–control study to quantitatively identify the risk factors related to school dropout. The results of this case–control study will provide input for a qualitative study with semi-structured interviews and homogenous focus groups. The results of the case–control study will also be discussed in the steering group, which consists of stakeholders from policy, practice and research, and with representatives from a youth organisation that provides support for school dropouts (expert group). Approval for conducting this study was granted by the Medical Ethics Committee of Maastricht University (METC 11-4-099, decision 22-08-2011).

### Mixed methods part 1: case-control study

The case–control study compares school dropouts with a control group with regard to medical, developmental, socioeconomic, environmental, psychosocial and personality-, lifestyle- and gender-related determinants. School dropouts are defined as people aged 18–24 who have received lower secondary education or less and are no longer in education or training [28]. They have had only a pre-primary, primary, lower secondary or short upper secondary education of less than two years [4,29].

#### Data collection

1.Compulsory education department

The Compulsory Education Departments (CED) of Eindhoven and Maastricht provided names, addresses and other information about the school careers of eligible participants. This information, collected during education, is not susceptible to recall bias. Based on this information, students were divided in a case (‘dropout’) or control group.

2.Preventive child & youth healthcare files

In the Netherlands, Preventive Child and Youth Healthcare (PCYH) doctors and specialised nurses offer routine health examinations and anticipatory guidance to all children between birth and 19 years [30,31]. They record the outcomes of the health examinations as well as information retrieved from different informants, such as parents, teachers and the young people themselves, in PCYH files. These files provide standardised longitudinal data on children’s health, psychosocial circumstances and functioning. This data is not susceptible to recall bias.

3.Questionnaire

The questionnaire will provide additional data on past and current health status, socioeconomic status, life events [32,33], gender [34,35] and personality traits such as rebelliousness [36,37], perceived control [38,39], neuroticism and extraversion [40,41] and social adequacy [42,43]. Personality traits are fairly stable and we assume that personality is not altered by school dropout. Because the last routine PCYH examination is given at the age of 14, the questionnaire refers to the age of 16 for substance use and behaviour.

### Sample size calculation

The power calculation for a retrospective study with a dichotomous outcome variable indicates that 290 cases would yield an 80% power to detect an odds ratio of 1.75 at a α-level of 5% for an exposure of 0.2 and a ratio of cases to controls of 1 [44,45]. In total, 580 participants need to be included: 290 cases and 290 controls. It is estimated that four times that number need to be contacted in order to collect information on 580 participants. Hence, approximately 2300 young adults will be contacted.

### Study population

#### Setting

In November 2011, the CED selected all eligible young adults aged 18–24 years who had received only lower secondary education or less and lived in Eindhoven. We performed a random sample among participants who had dropped out of school during the 2010–2011 school year (cases) and participants who still attended school or had graduated from at least upper secondary education during or at the end of the 2010–2011 school year (controls). We excluded cognitively impaired young people (IQ < 70), young people exempt from compulsory school attendance and young people with a lack of history in the PCYH files. In September 2012, we expanded the region to Maastricht and the villages around Eindhoven to enlarge the sample. The cities of Eindhoven and Maastricht are both located in the south of the Netherlands and have approximately 216,000 and 120,000 inhabitants, respectively [46].

#### Recruitment

We sent a paper questionnaire with study information and an informed consent to the selected participants. In addition, we asked for permission to approach them for an interview. Two reminders were sent. The second reminder included an internet questionnaire. Once we have expanded the region, selected participants will only receive a letter with study information and details for completing the online questionnaire. They will again be sent two reminders. More extensive information and details about the required consent can be found on the website. As an incentive, a raffle will be held among participants who fill in the questionnaire. They will receive cinema tickets. Young adults who do not return the questionnaire will be excluded from participation, but will be included in the non-responders analysis, which provides basic demographic information on the non-responders, such as age, sex and socioeconomic status.

### Statistical analyses

We will organise the information from the PCYH files with the International Classification of Functioning, Disability and Health for Children and Youth (ICF-CY) (Table 1) [47] and divide it over five timeframes. This is in line with the structure of the Dutch school system for early childhood (0–4 y and 4–8 y), middle childhood (8–12 y) and adolescence (12–16 y and 16–20 y) education [15]. The routine health examinations conducted as part of the Dutch PCYH system take place at regular set times and key moments in children’s development [31]. The ICF-CY provides a multi-perspective approach and emphasises the interactive nature between domains [47,48]. We will translate the combinations of and interactions between these domains into risk profiles for the early identification of school dropout.

**Table 1:** Determinants from the PCYH files in the ICF-CY model


**1. Disorder / Disease**	**4. Participation**
Congenital abnormality	All-day childcare
Physical illness	School
Mental illness	Sports / Club
Learning disability	Friends / Relationships
Pregnancy	Work
2. Symptoms*	**5. Environmental factors**
Internalising / Externalising behaviour	Ethnicity
Somatic complaints	Pregnancy / Childbirth
Social problems	Family composition
Sleeping difficulties	Parent–child relationship
Eating difficulties	Health of parents / siblings
Concentration difficulties	Parental education / profession
Learning difficulties	Social environment
Enuresis / Encopresis	Life event
3. Development*	Child abuse
Growth	Bullying (victim)
Motor	**6. Personal factors**
Speech / Language	Sex
Cognitive	Neonatal period
Social	Lifestyle
Sexual	Personality

*The ICF-CY model has been adapted for a better match with the life-course determinants from the youth health care files. ‘Activity’ was changed to ‘Development’, which indicates age-appropriate abilities in a life-course perspective.

We will use logistic analysis to relate potential predictors (disorders, symptoms, developmental, participatory, environmental and personal factors) to subsequent dropout. We will first conduct hierarchical analyses and separate checks on background characteristics such as gender and parental socioeconomic status to obtain insight into pathways that lead to dropout. This information reveals more recent (intermediate) characteristics in young adolescence such as developing disorders and symptoms. The focus on pathways will enable us to discover whether and how strongly adversities in young adulthood (e.g. hospital visits) affect school dropout, independent of the background characteristics during early life. Secondly, we examine profiles that combine life-course determinants to provide input for a tool to help identify risk profiles that may help predict pathways leading to school dropout. We use advanced techniques that allow the detection of optimal prediction models. These vary from a multiplicative interaction model (used for a stepwise approach) to advanced cluster and factor analyses. We will apply methods based on comparing areas under the curve (ROC) to find the profile and characteristics combinations with the best sensitivity and specificity, regarding the prediction of dropout. Depending on the measurement level of the variables, we use chi^2^-tests (categorical variable) or t-tests (continuous) to compare the demographic and socioeconomic characteristics of the responders and non-responders.

### Mixed methods part 2: qualitative study

This study will conduct semi-structured interviews using a topic list based on literature and findings from the case–control study to understand the lives of the young men and women participating. These young people, both cases and controls, who all have different socioeconomic backgrounds, will be interviewed until no new information emerges (theoretical saturation). This will maximise the depth and richness of the data and increase the validity of the results [49]. Marginally or less socially acceptable views are best assessed in individual interviews [49] and will therefore be used to explore the participants’ normative beliefs about gendered interaction with peers, parents and teachers. We will also stratify socioeconomic status. Male and female interviewers may interact differently with male and female participants and for this reason all four dyads will be included [50-52]. We will record and transcribe the interviews for analysis [53,54]. Interim analyses will be conducted by starting the analytical process during data collection, which will allow us to go back and refine questions, develop hypotheses and explore these in more depth [55]. We will furthermore read the data to identify emergent as well as anticipated themes and categories [56].

We will conduct six homogenous focus group sessions consisting of six to twelve people to explore a wide range of views held by parents and stakeholders [49]. A moderator will use an interview protocol based on the health-related determinant model for school dropout [57]. The focus group discussions aim to validate whether the themes found are recognisable. Also, they will explore possibilities for a tool to identify risk profiles for school dropout in the daily practice of public health [58]. The focus groups (which take approximately 90–120 minutes) will take place in the following order: (1) parents, (2) teachers and (3) PCYH professionals who have experience with the youths in the control group. We chose this order because it reflects the order of problem solving dimensions in daily practice. Next we will hold focus groups with (4) parents, (5) teachers and (6) PCYH professionals who have experience with the youth in the case group. This way information from previous interviews can be presented to the next groups [55]. Earlier findings and insights will be discussed in these last three focus groups with the aim of learning which issues were overlooked and which tools may be useful and feasible. We will record and transcribe the focus group interviews and an observer will take notes to describe the context and flow of the interview.

### Study population

For the interviews, we will make a selection based on the sex and socioeconomic status of the questionnaire responders (both cases and controls) who consented to being approached for an interview.

For the focus groups, we will ask the participants’ permission to contact their parents. We will guarantee heterogeneity by purposive sampling [59]. Teachers and PCYH professionals will be recruited through the existing network and snowball sampling. All travel expenses will be refunded.

### Qualitative analyses of statistical analysis

All interviews will be tape-recorded and transcribed verbatim and notes will be taken on the content of the individual interviews. We will read the notes and transcripts to gain a sense of the depth of the data and to collect and discuss ideas. We will apply researcher triangulation to base data collection, coding and analytic decisions on convergent validation [60]. We will analyse the transcripts thematically to identify and report patterns and categories in the data [49,61]. After organising the data into meaningful groups, we will construct themes by combining codes to overarching categories (axial coding). To validate the findings with other stakeholders (method triangulation), we will discuss these themes in the focus groups. The group is the unit of analysis. We will test the validity of the findings by comparing the responses given in the focus groups as well as the findings from the other methods of data collection (data, researcher and method triangulation).

## Discussion

The SIODO study aims to contribute to the reduction of school dropout and socioeconomic health differences using an integrative approach that is both broadening and deepening. This study has several unique characteristics. First, the case–control trial will be followed by a qualitative study with the aim of further interpreting and understanding the acquired information. Second, the determinants of school dropout will be viewed from a life-course perspective. The PCYH files providing longitudinal data from birth to adolescence will enable us to gain insight into the life-course pathways leading to school dropout. This information is not affected by retrospective bias. Third, all of the stakeholders involved will participate, including the young adults and their parents. This will create a better understanding of the pathways leading to dropout and will contribute to a prevention tool. PCYH professionals working in the field of school dropout and unauthorised school absence will be able to learn from the valuable experiences shared by the dropouts, their parents and professionals in other fields. Fourth, this study will explore self-assigned masculine and feminine characteristics and gendered beliefs and their association with dropout in more depth. The subject of gender is still underrepresented in public health research. Fifth, our study will specifically address socioeconomic status and its relation to school dropout and health. This study will particularly provide more in-depth information on how health and developmental problems in early life contribute to adolescent school dropout, thus adding to the knowledge about the under-studied selection perspectives on the (persisting and widening) socioeconomic inequalities in health. Finally, the availability of information on the non-responders in the PCYH files allows for a detailed analysis of possible selection biases.

The PCYH system is well-suited for detecting and monitoring youths and families at risk for dropout [62]. Rather than looking at early life symptoms from a dichotomous perspective (poor, good), we will assess symptoms that vary along a continuum from normal variations to problems, and finally disorders (with diagnoses) [15]. The focus on symptoms, rather than on diagnoses, may require a shift in the current medical model, in which therapy is only followed after a diagnosis has been given. Even without a diagnosis, certain symptoms have a great impact on the child’s development and quality of life [15,63]. School dropout is not an abrupt event, but a gradual process that often begins early in life. It is important that Dutch PCYH professionals, who offer routine health examinations and anticipatory guidance from birth, monitor social participation at each consultation, because joining sports, playing with friends or going to school is considered important for a healthy development. As school dropout has such a profound impact on an adolescent’s life and increases the risks of social exclusion, it is important that PCYH professionals are able to detect and monitor youths and families at risk for dropout, also in cases where no disorder has been diagnosed. SIODO aims to contribute to effective early childhood interventions for preventing young people from dropping out of school.

This study also has some limitations that need to be addressed. First, the data described in the PCYH files were not collected for the purpose of this study, so important information might be missing. However, the information in the PCYH files is not retrospectively biased, which means that this disadvantage is also one of the study’s strengths. Secondly, not all data in the PCYH files will be complete and these files will have been compiled by many different PCYH professionals, each with their own interpretations and descriptions of certain situations, symptoms and life-course determinants. However, because the PCYH professionals work with this information in their daily practice, the ecological validity of our findings will likely be high and provide added value for developing the prevention tool. The collaboration between research, practice and policy will certainly enhance the study’s use for optimising daily practice. Finally, participants in both the case and control groups had already been brought under the attention of the municipal CED. This may be why we had difficulties including participants who returned the completed questionnaire. We therefore included another Dutch region to increase the number of participants. For financial reasons, we proceeded with online questionnaires only.

The SIODO study will use a life-course perspective, the ICF-CY model to group the determinants and a mixed-methods design. In this respect, the SIODO study is innovative because it both broadens and deepens the study of the health-related determinants of school dropout. It examines how these determinants contribute to socioeconomic and gender differences in health and contributes to the development of a tool that can be used in public health practice to tackle the problem of school dropout at its roots.

### Ethics

Approval for conducting this study was granted by the Medical Ethics Committee of Maastricht University.

## Abbreviations

SIODO: Stay in or drop out; PCYH: Preventive child & youth healthcare; ICF: International classification of functioning, disability and health; ICF-CY: International classification of functioning, disability and health for children and youth; CED: Compulsory education department; SES: Socioeconomic status.

## Competing interests

The authors declare that they have no competing interests.

## Authors’ contributions

MT designed the study, wrote the protocol, conducted literature searches and drafted the manuscript. IG conducted literature searches and drafted the manuscript. FF, HB and PV participated in the design of the study and the protocol and critically revised the manuscript for important intellectual content. HB conducted the sample size calculation. All authors read and approved the final manuscript.

## Authors’ information

Ilse van Griensven and Marie-José Theunissen shared first authorship.

## Pre-publication history

The pre-publication history for this paper can be accessed here:

http://www.biomedcentral.com/1471-2458/12/1033/prepub
